# Dichloridoocta­kis(4-chloro­benz­yl)di-μ_2_-hydroxido-di-μ_3_-oxido-tetra­tin(IV) toluene solvate

**DOI:** 10.1107/S1600536809015128

**Published:** 2009-04-30

**Authors:** Kong Mun Lo, Seik Weng Ng

**Affiliations:** a Department of Chemistry, University of Malaya, 50603 Kuala Lumpur, Malaysia

## Abstract

The title stannoxane is a toluene-solvated dimer, [Sn_4_(C_7_H_6_Cl)_8_Cl_2_O_2_(OH)_2_]·C_7_H_8_, the tetra­nuclear mol­ecule lying across a center of inversion. The Sn_4_O_4_ framework, whose two independent Sn atoms show trigonal bipyramidal coordination, is essentially planar (r.m.s deviation = 0.02 Å). One of the two chloro­benzyl groups of the chloridodiorganyltin unit is disordered over two positions with the chloro­phenyl residue refined over two positions in a 50:50 ratio. The solvent mol­ecule is disordered about a twofold axis.

## Related literature

The distannoxane is a hydrolysed product of di(4-chloro­benz­yl)dichloridotin(IV); for the synthesis of the organotin compound by the direct reaction of 4-chloro­benzyl chloride and metallic tin, see: Shishido *et al.* (1961 ▶). For octa­benzyl­dichloridodi-μ_2_-hydroxo-di-μ_3_-oxo-tetra­tin, which crystallizes as a toluene disolvate, see: Mohamed *et al.* (2004 ▶). For octa­(4-methyl­benz­yl)dichloridodi-μ_2_-hydroxo-di-μ_3_-oxo-tetra­tin, see: Wang *et al.* (2007 ▶).
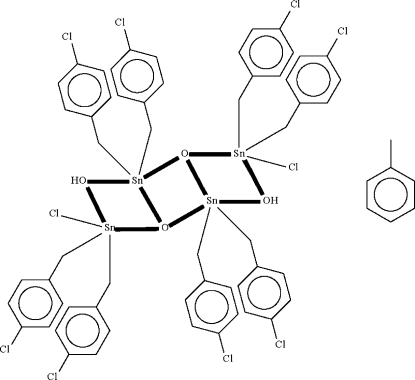


## Experimental

### 

#### Crystal data

[Sn_4_(C_7_H_6_Cl)_8_Cl_2_O_2_(OH)_2_]·C_7_H_8_
*M* *_r_* = 1708.35
Monoclinic, 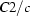

*a* = 26.6531 (3) Å
*b* = 10.8342 (1) Å
*c* = 25.8025 (3) Å
β = 121.546 (1)°
*V* = 6349.8 (1) Å^3^
*Z* = 4
Mo *K*α radiation
μ = 2.02 mm^−1^
*T* = 100 K
0.36 × 0.12 × 0.08 mm

#### Data collection

Bruker SMART APEX diffractometer
Absorption correction: multi-scan (*SADABS*; Sheldrick, 1996 ▶) *T*_min_ = 0.564, *T*_max_ = 0.746 (expected range = 0.643–0.851)
21584 measured reflections
7288 independent reflections
6556 reflections with *I* > 2σ(*I*)
*R*_int_ = 0.025

#### Refinement

*R*[*F* ^2^ > 2σ(*F* ^2^)] = 0.032
*wR*(*F* ^2^) = 0.118
*S* = 0.97
7288 reflections
389 parameters
127 restraints
H-atom parameters constrained
Δρ_max_ = 1.08 e Å^−3^
Δρ_min_ = −1.53 e Å^−3^

### 

Data collection: *APEX2* (Bruker, 2008 ▶); cell refinement: *SAINT* (Bruker, 2008 ▶); data reduction: *SAINT*; program(s) used to solve structure: *SHELXS97* (Sheldrick, 2008 ▶); program(s) used to refine structure: *SHELXL97* (Sheldrick, 2008 ▶); molecular graphics: *X-SEED* (Barbour, 2001 ▶); software used to prepare material for publication: *publCIF* (Westrip, 2009 ▶).

## Supplementary Material

Crystal structure: contains datablocks global, I. DOI: 10.1107/S1600536809015128/tk2437sup1.cif

Structure factors: contains datablocks I. DOI: 10.1107/S1600536809015128/tk2437Isup2.hkl

Additional supplementary materials:  crystallographic information; 3D view; checkCIF report

## Figures and Tables

**Fig. 1.**
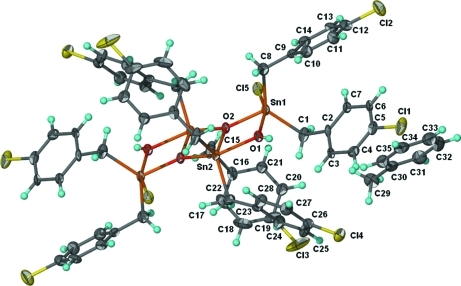
Thermal ellipsoid plot (Barbour, 2001) of [Sn2Cl(C7H6Cl)4O(OH)]2.C7H8 at the 70% probability level. Hydrogen atoms are drawn as spheres of arbitrary radius. For reasons of clarity, the disorder in the chlorophenyl group is not shown nor is the disordered solvent molecule. Unlabelled atoms are related to the labelled atoms by a centre of inversion.

## supplementary crystallographic information

### Experimental

Di(4-chlorobenzyl)tin dichloride (1 g, 2.2 mmol) (Shishido *et al.*, 1961) was recrystallized from toluene in the presence of excess pyridine (1 ml) to give well formed, colorless crystals.

### Refinement

Carbon-bound H-atoms were placed in calculated positions (C–H 0.95–0.99 Å) and were included in the refinement in the riding model approximation with *U*(H) set to 1.2–1.5*U*(C). The oxygen-bound H-atom was similarly treated (O–H 0.84 Å, *U*(H) 1.5*U*(O)).

One of the four chlorobenzyl groups, with the methylene-C1 atom, has its chlorophenyl residue disordered over two positions. As the occupancy refined to nearly 50:50, the occupancy was fixed as exactly 50:50. The phenyl ring was refined as a rigid hexagon of 1.39 Å. The temperature factors of the two chlorophenyl portions were restrained to be nearly isotropic.

The toluene molecule is disordered about a 2-fold axis; the phenyl part was also refined as a rigid hexagon, and the temperature factors of the carbon atoms were similarly restrained to be nearly isotropic.

The final difference Fourier map had a peak near Cl3 and a hole near the Sn2 atom.

### Crystal data

**Table:**

[Sn_4_(C_7_H_6_Cl)_8_Cl_2_O_2_(OH)_2_]·C_7_H_8_	*F*(000) = 3352
*M**_r_* = 1708.35	*D*_x_ = 1.787 Mg m^−^^3^
Monoclinic, *C*2/*c*	Mo *K*α radiation, λ = 0.71073 Å
Hall symbol: -C 2yc	Cell parameters from 9991 reflections
*a* = 26.6531 (3) Å	θ = 2.5–28.3°
*b* = 10.8342 (1) Å	µ = 2.02 mm^−^^1^
*c* = 25.8025 (3) Å	*T* = 100 K
β = 121.546 (1)°	Block, colorless
*V* = 6349.8 (1) Å^3^	0.36 × 0.12 × 0.08 mm
*Z* = 4	

### Data collection

**Table:**

Bruker SMART APEX diffractometer	7288 independent reflections
Radiation source: fine-focus sealed tube	6556 reflections with *I* > 2σ(*I*)
graphite	*R*_int_ = 0.025
ω scans	θ_max_ = 27.5°, θ_min_ = 1.8°
Absorption correction: multi-scan (*SADABS*; Sheldrick, 1996)	*h* = −34→34
*T*_min_ = 0.564, *T*_max_ = 0.746	*k* = −14→14
21584 measured reflections	*l* = −33→33

### Refinement

**Table:**

Refinement on *F*^2^	Primary atom site location: structure-invariant direct methods
Least-squares matrix: full	Secondary atom site location: difference Fourier map
*R*[*F*^2^ > 2σ(*F*^2^)] = 0.032	Hydrogen site location: inferred from neighbouring sites
*wR*(*F*^2^) = 0.118	H-atom parameters constrained
*S* = 0.97	*w* = 1/[σ^2^(*F*_o_^2^) + (0.1*P*)^2^] where *P* = (*F*_o_^2^ + 2*F*_c_^2^)/3
7288 reflections	(Δ/σ)_max_ = 0.001
389 parameters	Δρ_max_ = 1.08 e Å^−^^3^
127 restraints	Δρ_min_ = −1.53 e Å^−^^3^

### Fractional atomic coordinates and isotropic or equivalent isotropic displacement parameters (Å^2^)

**Table:**

	*x*	*y*	*z*	*U*_iso_*/*U*_eq_	Occ. (<1)
Sn1	0.327847 (9)	0.98788 (2)	0.507504 (10)	0.01694 (9)	
Sn2	0.275327 (9)	0.70201 (2)	0.456210 (9)	0.01484 (9)	
Cl1	0.44736 (9)	1.2278 (2)	0.33532 (10)	0.0402 (5)	0.50
Cl1'	0.47812 (11)	1.3027 (2)	0.39056 (13)	0.0466 (6)	0.50
Cl2	0.56752 (4)	1.39884 (11)	0.58933 (5)	0.0421 (3)	
Cl3	0.42907 (6)	0.44291 (13)	0.75877 (5)	0.0566 (4)	
Cl4	0.27115 (4)	0.87250 (9)	0.18192 (4)	0.0302 (2)	
Cl5	0.29674 (4)	1.10490 (8)	0.56878 (4)	0.02368 (18)	
O1	0.33270 (10)	0.8384 (2)	0.45170 (10)	0.0201 (5)	
H2	0.3515	0.8354	0.4338	0.030*	
O2	0.27917 (10)	0.8466 (2)	0.50928 (10)	0.0167 (4)	
C1	0.28989 (16)	1.1114 (4)	0.43078 (17)	0.0255 (8)	
H1A	0.2541	1.0731	0.3966	0.031*	0.50
H1B	0.2780	1.1888	0.4419	0.031*	0.50
H1C	0.2582	1.0675	0.3949	0.031*	0.50
H1D	0.2719	1.1825	0.4392	0.031*	0.50
C2	0.3321 (3)	1.1416 (7)	0.4104 (3)	0.019 (3)	0.50
C3	0.3242 (2)	1.0776 (6)	0.3600 (3)	0.0192 (17)	0.50
H3	0.2944	1.0165	0.3413	0.023*	0.50
C4	0.3600 (2)	1.1032 (5)	0.3369 (2)	0.0224 (14)	0.50
H4	0.3546	1.0595	0.3024	0.027*	0.50
C5	0.4036 (2)	1.1927 (6)	0.3643 (3)	0.0240 (18)	0.50
C6	0.4115 (2)	1.2566 (5)	0.4148 (3)	0.0246 (19)	0.50
H6	0.4413	1.3178	0.4335	0.030*	0.50
C7	0.3757 (3)	1.2310 (6)	0.4378 (3)	0.0190 (18)	0.50
H7	0.3811	1.2747	0.4723	0.023*	0.50
C2'	0.3344 (3)	1.1548 (7)	0.4162 (3)	0.017 (3)	0.50
C3'	0.3450 (3)	1.0990 (6)	0.3743 (3)	0.0242 (19)	0.50
H3'	0.3220	1.0302	0.3514	0.029*	0.50
C4'	0.3891 (3)	1.1441 (6)	0.3660 (3)	0.0277 (18)	0.50
H4'	0.3964	1.1060	0.3374	0.033*	0.50
C5'	0.4227 (2)	1.2449 (6)	0.3996 (3)	0.028 (2)	0.50
C6'	0.4121 (2)	1.3006 (5)	0.4415 (3)	0.0279 (17)	0.50
H6'	0.4350	1.3695	0.4645	0.033*	0.50
C7'	0.3679 (3)	1.2556 (6)	0.4498 (3)	0.0177 (18)	0.50
H7'	0.3607	1.2936	0.4784	0.021*	0.50
C8	0.41833 (15)	0.9705 (3)	0.57839 (17)	0.0237 (7)	
H8A	0.4206	0.9632	0.6178	0.028*	
H8B	0.4343	0.8933	0.5720	0.028*	
C9	0.45657 (9)	1.07721 (16)	0.58191 (10)	0.0195 (6)	
C10	0.49762 (10)	1.06170 (16)	0.56468 (11)	0.0251 (7)	
H10	0.5022	0.9833	0.5512	0.030*	
C11	0.53194 (9)	1.1609 (2)	0.56725 (11)	0.0270 (8)	
H11	0.5600	1.1503	0.5555	0.032*	
C12	0.52521 (9)	1.27552 (18)	0.58704 (12)	0.0286 (8)	
C13	0.48416 (10)	1.29103 (16)	0.60427 (11)	0.0253 (7)	
H13	0.4796	1.3694	0.6178	0.030*	
C14	0.44984 (9)	1.19187 (19)	0.60170 (11)	0.0219 (7)	
H14	0.4218	1.2025	0.6135	0.026*	
C15	0.34351 (17)	0.5660 (4)	0.49764 (18)	0.0281 (8)	
H15A	0.3291	0.4888	0.4737	0.034*	
H15B	0.3777	0.5950	0.4956	0.034*	
C16	0.36408 (10)	0.5374 (2)	0.56319 (8)	0.0217 (7)	
C17	0.40371 (11)	0.61529 (19)	0.60939 (11)	0.0465 (13)	
H17	0.4170	0.6882	0.5997	0.056*	
C18	0.42397 (12)	0.5865 (2)	0.66972 (10)	0.0543 (15)	
H18	0.4511	0.6398	0.7013	0.065*	
C19	0.40460 (11)	0.4799 (2)	0.68384 (7)	0.0331 (9)	
C20	0.36497 (10)	0.40193 (18)	0.63764 (10)	0.0232 (7)	
H20	0.3517	0.3290	0.6473	0.028*	
C21	0.34471 (9)	0.43068 (19)	0.57732 (8)	0.0218 (7)	
H21	0.3176	0.3774	0.5457	0.026*	
C22	0.20707 (16)	0.7021 (4)	0.36242 (16)	0.0257 (8)	
H22A	0.1921	0.6167	0.3505	0.031*	
H22B	0.1742	0.7537	0.3575	0.031*	
C23	0.22529 (9)	0.7490 (2)	0.31922 (9)	0.0198 (7)	
C24	0.19185 (8)	0.8399 (2)	0.27705 (10)	0.0253 (7)	
H24	0.1590	0.8749	0.2768	0.030*	
C25	0.20642 (10)	0.8794 (2)	0.23531 (9)	0.0274 (8)	
H25	0.1836	0.9415	0.2065	0.033*	
C26	0.25443 (10)	0.8281 (2)	0.23573 (9)	0.0223 (7)	
C27	0.28787 (9)	0.7373 (2)	0.27789 (10)	0.0259 (7)	
H27	0.3207	0.7022	0.2782	0.031*	
C28	0.27330 (9)	0.6977 (2)	0.31963 (9)	0.0244 (7)	
H28	0.2962	0.6356	0.3484	0.029*	
C29	0.4229 (5)	0.9306 (13)	0.2428 (6)	0.033 (2)	0.50
H29A	0.4038	1.0072	0.2436	0.050*	0.50
H29B	0.4315	0.8792	0.2777	0.050*	0.50
H29C	0.3967	0.8858	0.2050	0.050*	0.50
C30	0.47904 (19)	0.9607 (5)	0.2457 (3)	0.0256 (15)	0.50
C31	0.4921 (3)	1.0827 (4)	0.2406 (3)	0.032 (2)	0.50
H31	0.4652	1.1465	0.2351	0.039*	0.50
C32	0.5444 (3)	1.1115 (4)	0.2438 (3)	0.043 (2)	0.50
H32	0.5533	1.1948	0.2403	0.052*	0.50
C33	0.5837 (2)	1.0182 (6)	0.2519 (3)	0.038 (2)	0.50
H33	0.6195	1.0378	0.2541	0.046*	0.50
C34	0.5707 (2)	0.8962 (5)	0.2570 (4)	0.036 (3)	0.50
H34	0.5976	0.8324	0.2625	0.043*	0.50
C35	0.5183 (2)	0.8675 (4)	0.2538 (3)	0.0278 (16)	0.50
H35	0.5094	0.7841	0.2573	0.033*	0.50

### Atomic displacement parameters (Å^2^)

**Table:**

	*U*^11^	*U*^22^	*U*^33^	*U*^12^	*U*^13^	*U*^23^
Sn1	0.01971 (14)	0.01661 (14)	0.01916 (14)	−0.00310 (8)	0.01341 (11)	0.00117 (8)
Sn2	0.01654 (13)	0.01655 (14)	0.01512 (13)	0.00008 (8)	0.01083 (10)	0.00288 (8)
Cl1	0.0258 (9)	0.0584 (13)	0.0445 (12)	0.0056 (9)	0.0240 (9)	0.0273 (10)
Cl1'	0.0477 (12)	0.0421 (12)	0.0787 (17)	0.0062 (9)	0.0528 (13)	0.0182 (11)
Cl2	0.0310 (5)	0.0405 (6)	0.0516 (6)	−0.0154 (4)	0.0194 (5)	0.0024 (5)
Cl3	0.0672 (8)	0.0566 (8)	0.0172 (5)	0.0364 (6)	0.0022 (5)	−0.0001 (5)
Cl4	0.0372 (5)	0.0391 (5)	0.0220 (4)	−0.0055 (4)	0.0209 (4)	0.0018 (4)
Cl5	0.0323 (4)	0.0210 (4)	0.0269 (4)	−0.0033 (3)	0.0217 (4)	−0.0027 (3)
O1	0.0228 (12)	0.0213 (12)	0.0204 (12)	−0.0058 (10)	0.0141 (10)	−0.0004 (10)
O2	0.0211 (11)	0.0176 (11)	0.0178 (11)	−0.0034 (9)	0.0145 (9)	0.0006 (9)
C1	0.0288 (18)	0.0257 (19)	0.0308 (19)	0.0052 (14)	0.0217 (16)	0.0083 (15)
C2	0.023 (5)	0.009 (4)	0.025 (5)	0.000 (4)	0.012 (4)	0.008 (4)
C3	0.024 (4)	0.019 (4)	0.018 (4)	−0.010 (3)	0.013 (3)	0.000 (3)
C4	0.023 (3)	0.029 (4)	0.017 (3)	−0.001 (3)	0.011 (3)	0.001 (3)
C5	0.017 (3)	0.028 (4)	0.029 (4)	0.005 (3)	0.013 (3)	0.014 (4)
C6	0.022 (4)	0.023 (4)	0.023 (4)	−0.009 (3)	0.007 (3)	0.010 (3)
C7	0.027 (4)	0.016 (4)	0.017 (4)	−0.005 (3)	0.014 (3)	−0.001 (3)
C2'	0.025 (6)	0.019 (5)	0.017 (4)	0.004 (4)	0.018 (4)	0.002 (4)
C3'	0.031 (5)	0.021 (4)	0.023 (4)	−0.004 (3)	0.016 (4)	−0.003 (3)
C4'	0.037 (5)	0.032 (4)	0.028 (4)	0.006 (4)	0.026 (4)	−0.002 (4)
C5'	0.028 (4)	0.033 (4)	0.035 (5)	0.004 (3)	0.024 (4)	0.010 (4)
C6'	0.023 (3)	0.032 (4)	0.026 (4)	0.003 (3)	0.012 (3)	0.004 (3)
C7'	0.024 (4)	0.022 (4)	0.012 (3)	−0.005 (3)	0.013 (3)	0.001 (3)
C8	0.0234 (17)	0.0237 (18)	0.0258 (18)	−0.0008 (14)	0.0141 (15)	0.0019 (14)
C9	0.0186 (15)	0.0239 (17)	0.0131 (14)	−0.0027 (13)	0.0061 (12)	0.0018 (13)
C10	0.0263 (17)	0.0274 (19)	0.0233 (17)	−0.0002 (15)	0.0140 (15)	0.0007 (15)
C11	0.0202 (16)	0.032 (2)	0.0310 (19)	−0.0012 (15)	0.0152 (15)	0.0036 (16)
C12	0.0171 (16)	0.036 (2)	0.0262 (19)	−0.0058 (15)	0.0064 (14)	0.0020 (16)
C13	0.0201 (16)	0.0267 (19)	0.0206 (17)	−0.0071 (13)	0.0047 (14)	−0.0063 (14)
C14	0.0202 (16)	0.0261 (18)	0.0179 (16)	−0.0005 (13)	0.0090 (13)	−0.0009 (13)
C15	0.0310 (19)	0.0249 (19)	0.041 (2)	0.0145 (15)	0.0271 (17)	0.0182 (16)
C16	0.0155 (15)	0.0196 (17)	0.0331 (19)	0.0043 (13)	0.0148 (14)	0.0068 (14)
C17	0.027 (2)	0.022 (2)	0.055 (3)	−0.0061 (16)	−0.0030 (19)	0.015 (2)
C18	0.043 (3)	0.019 (2)	0.043 (3)	−0.0004 (18)	−0.017 (2)	−0.0012 (19)
C19	0.032 (2)	0.031 (2)	0.0193 (18)	0.0152 (17)	0.0018 (16)	0.0017 (16)
C20	0.0198 (16)	0.0273 (19)	0.0231 (17)	0.0038 (14)	0.0118 (14)	0.0055 (14)
C21	0.0221 (16)	0.0234 (17)	0.0222 (16)	−0.0017 (13)	0.0132 (14)	−0.0005 (14)
C22	0.0193 (16)	0.042 (2)	0.0158 (16)	−0.0063 (15)	0.0093 (14)	0.0031 (15)
C23	0.0196 (15)	0.0276 (18)	0.0119 (14)	−0.0072 (13)	0.0081 (12)	−0.0032 (13)
C24	0.0271 (17)	0.0284 (19)	0.0252 (18)	0.0011 (15)	0.0171 (15)	0.0019 (15)
C25	0.0343 (19)	0.0260 (19)	0.0236 (18)	0.0033 (15)	0.0163 (16)	0.0077 (15)
C26	0.0284 (17)	0.0239 (18)	0.0172 (16)	−0.0084 (14)	0.0138 (14)	−0.0050 (13)
C27	0.0252 (17)	0.031 (2)	0.0267 (18)	0.0005 (15)	0.0172 (15)	0.0001 (16)
C28	0.0260 (18)	0.0260 (19)	0.0231 (18)	0.0028 (14)	0.0141 (15)	0.0022 (14)
C29	0.029 (4)	0.031 (5)	0.039 (5)	0.001 (4)	0.017 (4)	−0.003 (4)
C30	0.026 (3)	0.025 (4)	0.018 (3)	0.001 (3)	0.006 (3)	0.002 (3)
C31	0.035 (5)	0.026 (3)	0.031 (6)	0.003 (4)	0.015 (5)	−0.001 (3)
C32	0.058 (5)	0.036 (4)	0.032 (4)	−0.013 (4)	0.021 (4)	0.002 (3)
C33	0.033 (4)	0.050 (5)	0.027 (4)	−0.008 (4)	0.013 (3)	0.003 (4)
C34	0.038 (6)	0.036 (6)	0.021 (4)	−0.003 (4)	0.007 (4)	−0.002 (4)
C35	0.033 (4)	0.023 (4)	0.025 (3)	0.000 (3)	0.014 (4)	0.000 (3)

### Geometric parameters (Å, °)

**Table:**

Sn1—O2	2.023 (2)	C10—H10	0.9500
Sn1—C8	2.140 (4)	C11—C12	1.3900
Sn1—C1	2.154 (4)	C11—H11	0.9500
Sn1—O1	2.215 (2)	C12—C13	1.3900
Sn1—Cl5	2.4834 (8)	C13—C14	1.3900
Sn2—O2	2.047 (2)	C13—H13	0.9500
Sn2—O2^i^	2.129 (2)	C14—H14	0.9500
Sn2—C22	2.135 (3)	C15—C16	1.512 (4)
Sn2—C15	2.141 (3)	C15—H15A	0.9900
Sn2—O1	2.173 (2)	C15—H15B	0.9900
Cl1—C5	1.725 (4)	C16—C17	1.3900
Cl1'—C5'	1.730 (4)	C16—C21	1.3900
Cl2—C12	1.7292 (17)	C17—C18	1.3900
Cl3—C19	1.733 (2)	C17—H17	0.9500
Cl4—C26	1.7335 (16)	C18—C19	1.3900
O1—H2	0.8400	C18—H18	0.9500
O2—Sn2^i^	2.129 (2)	C19—C20	1.3900
C1—C2'	1.495 (5)	C20—C21	1.3900
C1—C2	1.505 (4)	C20—H20	0.9500
C1—H1A	0.9900	C21—H21	0.9500
C1—H1B	0.9900	C22—C23	1.518 (4)
C1—H1C	0.9900	C22—H22A	0.9900
C1—H1D	0.9901	C22—H22B	0.9900
C2—C3	1.3900	C23—C24	1.3900
C2—C7	1.3900	C23—C28	1.3900
C3—C4	1.3900	C24—C25	1.3900
C3—H3	0.9500	C24—H24	0.9500
C4—C5	1.3900	C25—C26	1.3900
C4—H4	0.9500	C25—H25	0.9500
C5—C6	1.3900	C26—C27	1.3900
C6—C7	1.3900	C27—C28	1.3900
C6—H6	0.9500	C27—H27	0.9500
C7—H7	0.9500	C28—H28	0.9500
C2'—C3'	1.3900	C29—C30	1.493 (10)
C2'—C7'	1.3900	C29—H29A	0.9800
C3'—C4'	1.3900	C29—H29B	0.9800
C3'—H3'	0.9500	C29—H29C	0.9800
C4'—C5'	1.3900	C30—C31	1.3900
C4'—H4'	0.9500	C30—C35	1.3900
C5'—C6'	1.3900	C31—C32	1.3900
C6'—C7'	1.3900	C31—H31	0.9500
C6'—H6'	0.9500	C32—C33	1.3900
C7'—H7'	0.9500	C32—H32	0.9500
C8—C9	1.512 (4)	C33—C34	1.3900
C8—H8A	0.9900	C33—H33	0.9500
C8—H8B	0.9900	C34—C35	1.3900
C9—C10	1.3900	C34—H34	0.9500
C9—C14	1.3900	C35—H35	0.9500
C10—C11	1.3900		
			
O2—Sn1—C8	111.30 (12)	C10—C9—C14	120.0
O2—Sn1—C1	119.52 (12)	C10—C9—C8	120.45 (19)
C8—Sn1—C1	128.14 (14)	C14—C9—C8	119.54 (19)
O2—Sn1—O1	73.60 (8)	C9—C10—C11	120.0
C8—Sn1—O1	93.91 (12)	C9—C10—H10	120.0
C1—Sn1—O1	90.90 (12)	C11—C10—H10	120.0
O2—Sn1—Cl5	86.57 (6)	C10—C11—C12	120.0
C8—Sn1—Cl5	95.92 (10)	C10—C11—H11	120.0
C1—Sn1—Cl5	96.62 (10)	C12—C11—H11	120.0
O1—Sn1—Cl5	159.98 (6)	C13—C12—C11	120.0
O2—Sn2—O2^i^	73.49 (10)	C13—C12—Cl2	120.27 (14)
O2—Sn2—C22	118.21 (13)	C11—C12—Cl2	119.73 (14)
O2^i^—Sn2—C22	96.37 (11)	C14—C13—C12	120.0
O2—Sn2—C15	117.40 (14)	C14—C13—H13	120.0
O2^i^—Sn2—C15	101.99 (11)	C12—C13—H13	120.0
C22—Sn2—C15	124.29 (16)	C13—C14—C9	120.0
O2—Sn2—O1	74.04 (8)	C13—C14—H14	120.0
O2^i^—Sn2—O1	147.53 (9)	C9—C14—H14	120.0
C22—Sn2—O1	98.35 (11)	C16—C15—Sn2	114.6 (2)
C15—Sn2—O1	93.30 (12)	C16—C15—H15A	108.6
Sn2—O1—Sn1	100.39 (9)	Sn2—C15—H15A	108.6
Sn2—O1—H2	129.8	C16—C15—H15B	108.6
Sn1—O1—H2	129.8	Sn2—C15—H15B	108.6
Sn1—O2—Sn2	111.85 (9)	H15A—C15—H15B	107.6
Sn1—O2—Sn2^i^	141.49 (12)	C17—C16—C21	120.0
Sn2—O2—Sn2^i^	106.51 (10)	C17—C16—C15	120.2 (2)
C2'—C1—Sn1	112.0 (4)	C21—C16—C15	119.8 (2)
C2—C1—Sn1	112.2 (4)	C16—C17—C18	120.0
C2'—C1—H1A	115.4	C16—C17—H17	120.0
C2—C1—H1A	109.2	C18—C17—H17	120.0
Sn1—C1—H1A	109.2	C19—C18—C17	120.0
C2'—C1—H1B	102.9	C19—C18—H18	120.0
C2—C1—H1B	109.2	C17—C18—H18	120.0
Sn1—C1—H1B	109.2	C18—C19—C20	120.0
H1A—C1—H1B	107.9	C18—C19—Cl3	120.66 (16)
C2'—C1—H1C	108.4	C20—C19—Cl3	119.34 (16)
C2—C1—H1C	101.8	C21—C20—C19	120.0
Sn1—C1—H1C	108.8	C21—C20—H20	120.0
C2'—C1—H1D	110.5	C19—C20—H20	120.0
C2—C1—H1D	116.4	C20—C21—C16	120.0
Sn1—C1—H1D	109.3	C20—C21—H21	120.0
H1C—C1—H1D	107.8	C16—C21—H21	120.0
C3—C2—C7	120.0	C23—C22—Sn2	115.0 (2)
C3—C2—C1	116.4 (4)	C23—C22—H22A	108.5
C7—C2—C1	123.6 (4)	Sn2—C22—H22A	108.5
C2—C3—C4	120.0	C23—C22—H22B	108.5
C2—C3—H3	120.0	Sn2—C22—H22B	108.5
C4—C3—H3	120.0	H22A—C22—H22B	107.5
C5—C4—C3	120.0	C24—C23—C28	120.0
C5—C4—H4	120.0	C24—C23—C22	119.5 (2)
C3—C4—H4	120.0	C28—C23—C22	120.5 (2)
C4—C5—C6	120.0	C25—C24—C23	120.0
C4—C5—Cl1	120.6 (4)	C25—C24—H24	120.0
C6—C5—Cl1	119.4 (4)	C23—C24—H24	120.0
C7—C6—C5	120.0	C24—C25—C26	120.0
C7—C6—H6	120.0	C24—C25—H25	120.0
C5—C6—H6	120.0	C26—C25—H25	120.0
C6—C7—C2	120.0	C27—C26—C25	120.0
C6—C7—H7	120.0	C27—C26—Cl4	119.67 (13)
C2—C7—H7	120.0	C25—C26—Cl4	120.28 (13)
C3'—C2'—C7'	120.0	C26—C27—C28	120.0
C3'—C2'—C1	124.7 (4)	C26—C27—H27	120.0
C7'—C2'—C1	115.2 (4)	C28—C27—H27	120.0
C2'—C3'—C4'	120.0	C27—C28—C23	120.0
C2'—C3'—H3'	120.0	C27—C28—H28	120.0
C4'—C3'—H3'	120.0	C23—C28—H28	120.0
C5'—C4'—C3'	120.0	C31—C30—C35	120.0
C5'—C4'—H4'	120.0	C31—C30—C29	119.7 (7)
C3'—C4'—H4'	120.0	C35—C30—C29	120.3 (7)
C4'—C5'—C6'	120.0	C30—C31—C32	120.0
C4'—C5'—Cl1'	121.1 (4)	C30—C31—H31	120.0
C6'—C5'—Cl1'	118.9 (4)	C32—C31—H31	120.0
C5'—C6'—C7'	120.0	C31—C32—C33	120.0
C5'—C6'—H6'	120.0	C31—C32—H32	120.0
C7'—C6'—H6'	120.0	C33—C32—H32	120.0
C6'—C7'—C2'	120.0	C34—C33—C32	120.0
C6'—C7'—H7'	120.0	C34—C33—H33	120.0
C2'—C7'—H7'	120.0	C32—C33—H33	120.0
C9—C8—Sn1	114.0 (2)	C33—C34—C35	120.0
C9—C8—H8A	108.8	C33—C34—H34	120.0
Sn1—C8—H8A	108.8	C35—C34—H34	120.0
C9—C8—H8B	108.8	C34—C35—C30	120.0
Sn1—C8—H8B	108.8	C34—C35—H35	120.0
H8A—C8—H8B	107.6	C30—C35—H35	120.0
			
O2—Sn2—O1—Sn1	−2.43 (8)	C1—C2'—C7'—C6'	177.6 (7)
O2^i^—Sn2—O1—Sn1	−1.4 (2)	O2—Sn1—C8—C9	−176.63 (19)
C22—Sn2—O1—Sn1	114.63 (13)	C1—Sn1—C8—C9	15.2 (3)
C15—Sn2—O1—Sn1	−120.01 (13)	O1—Sn1—C8—C9	109.5 (2)
O2—Sn1—O1—Sn2	2.47 (9)	Cl5—Sn1—C8—C9	−88.0 (2)
C8—Sn1—O1—Sn2	113.53 (12)	Sn1—C8—C9—C10	−108.9 (2)
C1—Sn1—O1—Sn2	−118.14 (12)	Sn1—C8—C9—C14	70.2 (3)
Cl5—Sn1—O1—Sn2	−5.8 (3)	C14—C9—C10—C11	0.0
C8—Sn1—O2—Sn2	−90.57 (14)	C8—C9—C10—C11	179.1 (2)
C1—Sn1—O2—Sn2	78.73 (16)	C9—C10—C11—C12	0.0
O1—Sn1—O2—Sn2	−2.78 (10)	C10—C11—C12—C13	0.0
Cl5—Sn1—O2—Sn2	174.40 (10)	C10—C11—C12—Cl2	−179.31 (19)
C8—Sn1—O2—Sn2^i^	94.6 (2)	C11—C12—C13—C14	0.0
C1—Sn1—O2—Sn2^i^	−96.1 (2)	Cl2—C12—C13—C14	179.30 (19)
O1—Sn1—O2—Sn2^i^	−177.6 (2)	C12—C13—C14—C9	0.0
Cl5—Sn1—O2—Sn2^i^	−0.40 (18)	C10—C9—C14—C13	0.0
O2^i^—Sn2—O2—Sn1	−176.62 (18)	C8—C9—C14—C13	−179.1 (2)
C22—Sn2—O2—Sn1	−88.21 (14)	O2—Sn2—C15—C16	37.0 (3)
C15—Sn2—O2—Sn1	88.19 (15)	O2^i^—Sn2—C15—C16	−40.4 (3)
O1—Sn2—O2—Sn1	2.82 (10)	C22—Sn2—C15—C16	−146.8 (2)
O2^i^—Sn2—O2—Sn2^i^	0.0	O1—Sn2—C15—C16	110.8 (2)
C22—Sn2—O2—Sn2^i^	88.41 (14)	Sn2—C15—C16—C17	−80.2 (3)
C15—Sn2—O2—Sn2^i^	−95.18 (14)	Sn2—C15—C16—C21	101.8 (2)
O1—Sn2—O2—Sn2^i^	179.45 (12)	C21—C16—C17—C18	0.0
O2—Sn1—C1—C2'	−145.1 (3)	C15—C16—C17—C18	−177.9 (2)
C8—Sn1—C1—C2'	22.2 (4)	C16—C17—C18—C19	0.0
O1—Sn1—C1—C2'	−73.5 (3)	C17—C18—C19—C20	0.0
Cl5—Sn1—C1—C2'	125.1 (3)	C17—C18—C19—Cl3	−179.7 (2)
O2—Sn1—C1—C2	−137.2 (3)	C18—C19—C20—C21	0.0
C8—Sn1—C1—C2	30.1 (4)	Cl3—C19—C20—C21	179.7 (2)
O1—Sn1—C1—C2	−65.6 (3)	C19—C20—C21—C16	0.0
Cl5—Sn1—C1—C2	133.0 (3)	C17—C16—C21—C20	0.0
C2'—C1—C2—C3	−170 (5)	C15—C16—C21—C20	177.9 (2)
Sn1—C1—C2—C3	100.5 (4)	O2—Sn2—C22—C23	98.0 (3)
C2'—C1—C2—C7	7(5)	O2^i^—Sn2—C22—C23	172.7 (2)
Sn1—C1—C2—C7	−81.9 (5)	C15—Sn2—C22—C23	−78.1 (3)
C7—C2—C3—C4	0.0	O1—Sn2—C22—C23	21.7 (3)
C1—C2—C3—C4	177.7 (6)	Sn2—C22—C23—C24	−129.00 (19)
C2—C3—C4—C5	0.0	Sn2—C22—C23—C28	54.1 (3)
C3—C4—C5—C6	0.0	C28—C23—C24—C25	0.0
C3—C4—C5—Cl1	−178.6 (5)	C22—C23—C24—C25	−176.9 (2)
C4—C5—C6—C7	0.0	C23—C24—C25—C26	0.0
Cl1—C5—C6—C7	178.7 (5)	C24—C25—C26—C27	0.0
C5—C6—C7—C2	0.0	C24—C25—C26—Cl4	177.44 (18)
C3—C2—C7—C6	0.0	C25—C26—C27—C28	0.0
C1—C2—C7—C6	−177.5 (7)	Cl4—C26—C27—C28	−177.45 (18)
Sn1—C1—C2'—C3'	93.2 (5)	C26—C27—C28—C23	0.0
C2—C1—C2'—C7'	−178 (5)	C24—C23—C28—C27	0.0
Sn1—C1—C2'—C7'	−84.3 (4)	C22—C23—C28—C27	176.9 (2)
C7'—C2'—C3'—C4'	0.0	C35—C30—C31—C32	0.0
C1—C2'—C3'—C4'	−177.4 (7)	C29—C30—C31—C32	179.6 (9)
C2'—C3'—C4'—C5'	0.0	C30—C31—C32—C33	0.0
C3'—C4'—C5'—C6'	0.0	C31—C32—C33—C34	0.0
C3'—C4'—C5'—Cl1'	179.7 (5)	C32—C33—C34—C35	0.0
C4'—C5'—C6'—C7'	0.0	C33—C34—C35—C30	0.0
Cl1'—C5'—C6'—C7'	−179.7 (5)	C31—C30—C35—C34	0.0
C5'—C6'—C7'—C2'	0.0	C29—C30—C35—C34	−179.6 (9)
C3'—C2'—C7'—C6'	0.0		

Symmetry codes: (i) −*x*+1/2, −*y*+3/2, −*z*+1.
